# Human Stove

**Published:** 1875-02

**Authors:** 


### HUMAN STOVE.

THE heat of the body is derived from the food we eat; fats, oils and sweets give more heat than anything else. If all this heat could be confined to the body for twenty-four hours it would raise Fahrenheit's thermometer to 185° and all the flesh would be cooked. The colder the weather the more fats and sweets we must eat, and the warmer the climate the less the amount needed, and the people live mainly on fruits, vegetables and such other food as contains no oil and are rather acid than sweet, hence are cooling. Thus it is that the food of a latitude is adapted to the wants of the people who live there, the natural heat of the body being the same, 98°, in every part of the globe.

